# Adverse childhood experience categories and subjective cognitive decline in adulthood: an analysis of the Behavioral Risk Factor Surveillance System

**DOI:** 10.1515/jom-2022-0140

**Published:** 2022-11-08

**Authors:** Rachel M. Terry, Sadie E. Schiffmacher, Avery A. Dutcher, Julie M. Croff, Martina J. Jelley, Micah L. Hartwell

**Affiliations:** Oklahoma State University Center for Health Sciences, 1111 W 17th Street, Tulsa, OK 74107, USA; Oklahoma State University College of Osteopathic Medicine at the Cherokee Nation, Office of Medical Student Research, Tahlequah, OK, USA; Oklahoma State University Center for Health Sciences, Office of Medical Student Research, Tulsa, OK, USA; National Center for Wellness and Recovery, Tulsa, OK, USA; Oklahoma State University Center for Health Sciences, Center for Integrative Research on Childhood Adversity, Tulsa, OK, USA; and Oklahoma State University Center for Health Sciences, Center for Rural Health, Tulsa, OK, USA; Department of Internal Medicine, University of Oklahoma School of Community Medicine, Tulsa, OK, USA; Oklahoma State University Center for Health Sciences, Office of Medical Student Research, Tulsa, OK, USA; and Department of Psychiatry and Behavioral Sciences, Oklahoma State University Center for Health Sciences, Tulsa, OK, USA

**Keywords:** adverse childhood experience, Alzheimer’s disease, cognitive decline, dementia

## Abstract

**Context:**

Adverse childhood experiences (ACEs) negatively impact health outcomes later in life, in a dose–dependent relationship; however, little is known about the impact of the individual ACE categories and subjective cognitive decline (SCD) later in life.

**Objectives:**

The aim of this study was to determine the associations among the eight ACEs and SCD.

**Methods:**

We analyzed data from two cycles of the Behavioral Risk Factor Surveillance System (BRFSS; 2019–2020). We assessed the accumulation of ACEs and their association with SCD, and among individuals reporting only one ACE, we utilized logistic regression to compare the likelihood of reporting SCD and symptomology among the eight categories of adversity.

**Results:**

Among included respondents, 10.14% reported experiencing SCD. More ACEs were reported among those with SCD (mean, 2.61; SD, 2.56) compared to those without SCD (mean, 1.44; SD, 1.91). Those with higher ACE scores were significantly less likely to have spoken with a healthcare provider about their cognitive decline. Individuals reporting one ACE of either family mental illness, family substance abuse, family incarceration, emotional abuse, or physical abuse had significantly greater odds of reporting memory loss compared to individuals with no ACEs.

**Conclusions:**

Having multiple ACEs was significantly associated with higher odds of SCD and associated limitation of social activity and was inversely associated with getting help when it is needed. Further, many ACE categories were associated with SCD – a novel addition to the literature and the methodology utilized herein. Interventions focused on improving cognitive health and preventing cognitive decline should consider the potential role of ACEs among affected populations.

Declines in cognitive functioning impede maintenance of a healthy, active, and independent lifestyle. Subjective cognitive decline (SCD) ranges in severity from mild cognitive impairment to dementia. The net cost of care for the most severe cognitive declines (i.e., dementia) was nearly twice (1.75 times) as much as the costs of care for a person without dementia [1]. Dementia is one of the leading causes of care dependency and disability in old age globally [2]. In 2019, the World Health Organization (WHO) estimated that 55.2 million people were living with dementia worldwide (10.3 million people in America) [2]. Alzheimer’s disease is the leading cause of dementia [3]. Alzheimer’s disease impacted 6.2 million older Americans in 2021 [3]. Alzheimer’s diagnoses are estimated to grow to 13.8 million by 2060 [3]. As rates of dementia in the United States are estimated to increase, it is necessary to explore potential contributing factors of cognitive decline in order to establish preventative measures.

A growing body of literature has linked adverse childhood experiences (ACEs) to SCD [4–7]. SCD refers to an individual’s subjective experience of worsening or more frequent confusion or memory loss within the past 12 months. ACEs are potentially traumatic events that occur during childhood before the age of 18 [8]. ACEs include three domains of early life adversity: abuse, household dysfunction, and neglect [8]. The eight ACE categories are Family Mental Illness, Substance Abuse in the Household, Incarcerated Household Member, Parental Separation or Divorce, Intimate Partner Violence, Emotional Abuse, Physical Abuse, and Sexual Abuse [8]. ACEs have been linked to a variety of adverse physical and mental outcomes, including negative impacts on mental health, intellectual development, immune and metabolic function, and neurological development [9, 10]. Early life stress associated with ACEs may impact structural and functional neurological development, impacting cognitive functioning later in life [10]. Evidence points to cumulative effects of ACEs on SCD; however, there is a lack of studies investigating the category-specific effects of ACEs. Brown et al. [7] previously reported the associations of ACEs and SCD utilizing the 2019 Behavioral Risk Factor Surveillance System (BRFSS) data. However, within the Brown et al. [7] study, ACE categories were collapsed and did not account for multiple cross-category ACEs. Thus, our objectives were: (1) to extend the literature on ACEs and SCD including data from the 2020 BRFSS, which expanded to 28 states and Washington D.C. and which is the largest collection of ACEs to date; and (2) to investigate the impact of the 8 ACE categories on SCD by comparing respondents with SCD reporting only 1 ACE, stratified by categories, to individuals reporting no ACEs. Our study adds to the current literature on ACEs and SCD by expanding upon the findings of Brown et al. [7] – which categorized ACEs into three groups (sexual, physical/psychological, and environmental) with associations between all three groups and SCD.

## Methods

We performed a cross-sectional analysis of the Center for Disease Control and Prevention (CDC) 2019 and 2020 BRFSS. BRFSS is an annual survey of US adult residents, administered via landlines or cellular phones by the health departments of all 50 states, the District of Columbia, Puerto Rico, and Guam. The BRFSS Cognitive Decline Module was administered to individuals 45 years or older, thus our analysis excluded individuals under the age of 45. Institutional Review Board approval was sought and obtained for the BRFSS survey administration through the CDC.

### Adverse childhood experiences

The BRFSS ACE Module was adapted from the CDC-Kaiser ACE Scale to categorize the components of ACEs and consists of 11 questions evaluating adverse events in childhood before the age of 18 [8]. The eight ACEs assessed in this study include: Family Mental Illness, Family Substance Abuse, Family Incarceration, Parental Divorce, Intimate Partner Violence, Emotional Abuse, Physical Abuse, and Sexual Abuse. These experiences are further defined in Table 1. Each individual participant was assigned an ACE score of 0–11 based upon their answers to the ACE section in the BRFSS questionnaire. For analyses, we will categorize groups based on 0, 1–2, and 3+ ACEs.

### Subjective cognitive decline

SCD, the self-reported experience of worsening or more frequent confusion or memory loss within the past 12 months, was assessed based on participant responses to a 6-question BRFSS Cognitive Decline Module developed by the CDC. The Cognitive Decline Module questions measure the BRFSS respondents’ perception about any confusion or memory loss that is happening more or less often [11]. Survey respondents were first asked, “During the past 12 months, have you experienced confusion or memory loss that is happening more often or is getting worse?” If a respondent endorsed the initial item, he or she received five additional questions assessing the frequency of interference in daily living: (1) During the past 12 months, as a result of confusion or memory loss, how often have you given up day-to-day household activities or chores you did previously, such as cooking, cleaning, taking medications, driving, or paying bills?; (2) As a result of confusion or memory loss, how often do you need assistance with these day-to-day activities?; (3) When you need help with these day-to-day activities, how often are you able to get the help that you need?; (4) During the past 12 months, how often has confusion or memory loss interfered with your ability to work, volunteer, or engage in social activities outside the home?; and (5) Have you or anyone else discussed your confusion or memory loss with a healthcare professional? [11] Response frequency was dichotomized to *frequently* if the response was always or usually, and *infrequently* if the responses were sometimes, rarely, or never. Responses of unknown or refused were excluded from analyses.

### Sociodemographic variables

Sociodemographic variables included in this study were based on self-report and included age, sex, race, healthcare coverage, and education. Age was reported in three age groupings: 45–54, 55–64, and 65 years and older. Race was self-reported and included categories of White, Black, Asian, American Indian/Alaskan Native, Hispanic, and Other. Healthcare coverage was a binary variable based on the question of whether they had current healthcare coverage. Education was reported in four categories: less than high school, high school graduate, some college or technical school, and completed college or technical school.

### Statistical analysis

After combining the 2019 and 2020 BRFSS datasets, we adjusted sampling weights to account for the combined cycles, which were utilized with all analyses. We estimated the sample prevalence and population estimates for individuals experiencing SCD. Next, we estimated the mean ACEs among each group. Then, we reported the prevalence of individuals with frequent and infrequent disruption from SCD. To assess the association of cumulative ACEs on SCD symptoms, we utilized logistic regression to determine odds ratios and adjusted odds ratios – the latter controlling for age, sex, race, healthcare coverage, and education. Left off here Lastly, we selected respondents reporting only one ACE and stratified respondents by the ACE category they experienced. Then, we utilized logistic regression to assess differences in SCD among participants reporting only one ACE compared to those reporting no ACEs. Alpha was set at 0.05, and 95% confidence intervals were reported. Analyses were conducted utilizing Stata 16.1 (StataCorp, LLC, College Station, TX). This study was submitted for ethics review and was determined not to meet the requirements of human subjects by an Institutional Review Board; however, the BRFSS survey administration sought and was granted permission to administer the study through the CDC. This study adhered to the Strengthening the Reporting of Observational Studies in Epidemiology (STROBE) guidelines.

## Results

The response rates of the 2019 and 2020 BRFSS were 50.0 and 47.8%, respectively. Our final sample included 178,441 respondents representing a population estimate of 38,215,839. Among the respondents aged 45 and over, 10.14% (n=18,096; n=3,960,992) reported experiencing SCD. Mean ACE scores among participants reporting SCD were 2.61 (SD, 2.56) compared to an ACE score 1.44 (SD, 1.91) in participants not reporting SCD (Table 2).

**Figure 1:**
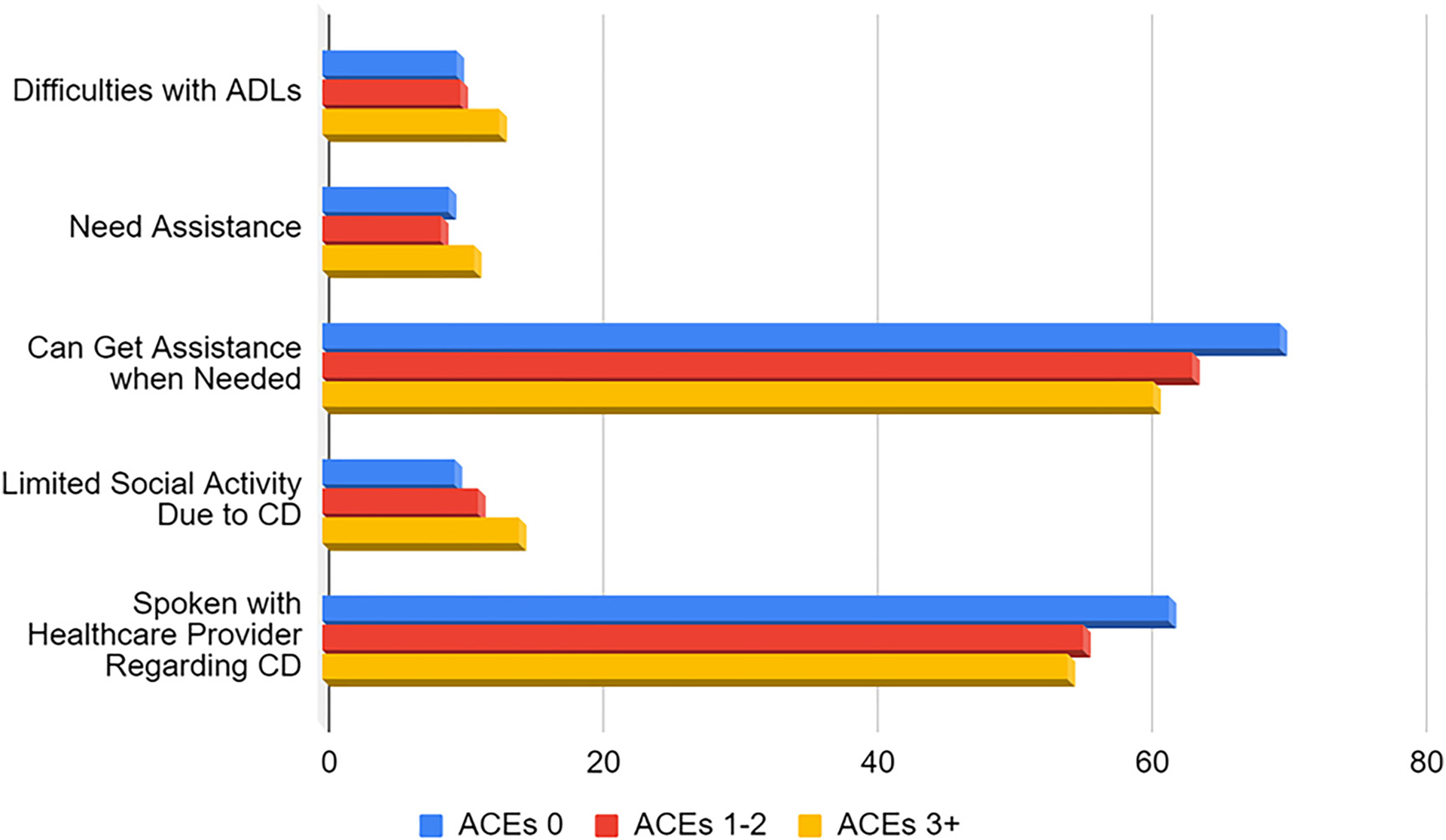
Prevalence of individuals experiencing cognitive decline reporting frequent impedance of life activities by number of adverse childhood events.

The distributions of individuals with SCD reporting frequent disruptions of day-to-day household activities, of needing assistance with those activities, receiving social support, and whether they have discussed memory loss were skewed to show negative disparities among those reporting more ACEs (Figure 1).

### Associations of ACEs and SCD

Logistic regression was utilized to determine the associations in SCD questions and the accumulation of ACEs compared to those reporting no ACEs (Table 3). Individuals with ACE scores greater than 0 were 1.59 (1–2 ACEs) and 3.58 (3+ ACEs) times more likely to report frequently experiencing memory loss (1–2 ACEs: AOR: 1.59; 95% CI: 1.43–1.76; 3+ ACEs: AOR: 3.58; 95% CI: 3.23–3.96). Individuals with ACE scores greater than 0 were also less likely to have spoken with a healthcare provider about their SCD (1–2 ACEs: AOR: 0.79; 95% CI: 0.65–0.97 and 3+ ACEs: AOR: 0.64; 95% CI: 0.52–0.79) than individuals with no ACEs. Individuals with ACE scores of three or more were 1.57 times more likely to report frequent difficulties with activities of daily living (ADLs; AOR: 1.57; 95% CI: 1.16–2.12), needing assistance with ADLs (AOR: 1.33; 95% CI: 0.96–1.86), and experiencing social limitations due to SCD (AOR: 1.71; 95% CI: 1.24–2.36) than individuals with no ACEs. Individuals with ACE scores of three or more were less likely to get help when needed with ADLs (AOR: 0.64; 95% CI: 0.43–0.93) compared to individuals with no ACEs. All relationships between SCD and cumulative ACEs are shown in Table 3.

### Associations of SCD and ACE category among individuals reporting one ACE

Compared to those who reported no ACEs, individuals with one ACE of family mental illness (AOR: 2.32; 95% CI: 3.74–3.09), family substance abuse (AOR: 1.55; 95% CI: 1.24–1.94), family incarceration (AOR: 2.09; 95% CI: 1.11–3.94), emotional abuse (AOR: 1.44; 95% CI: 1.19–1.74), and physical abuse (AOR: 1.51; 95% CI: 1.22–1.86) had higher odds of reporting memory loss (Table 4). Individuals with one ACE of parental divorce were less likely (AOR: 0.53; 95% CI: 0.30–0.94) to get help with ADLs when needed. Compared to individuals with no ACEs, individuals reporting sexual abuse (AOR: 0.24; 95% CI: 0.08–0.75) were less likely to experience social limitations compared to those with no ACEs. All relationships between SCD and ACE domains are shown in Table 4.

## Discussion

We found a statistically significant association between high ACE scores of three or more with frequent reports of SCD characteristics. Further, we found that compared to those who did not report any ACEs, individuals with one ACE of family mental illness, family substance abuse, family incarceration, emotional abuse, and physical abuse had higher odds of reporting memory loss. The models showed that individuals with one ACE of parental divorce were significantly less likely to get help with ADLs when needed, and individuals reporting one ACE of sexual abuse had a lower likelihood of reporting social limitations compared to others with no ACEs. Our study expands the current literature by: (1) investigating associations between SCD and each of the eight ACEs categories; (2) assessing the impact of ACE categories, singularly reported by individuals; and (3) combining data from the 2019 and 2020 BRFSS creating the widest dataset collected among US states. Our findings have important implications in identifying individuals at risk of developing SCD and the development of targeted interventions among people with an increased likelihood of having SCD.

Household dysfunction categories of family mental illness and family incarceration were the two ACE categories associated with the highest likelihood of experiencing memory loss. Parental mental illness has been shown to negatively impact cognitive development [12]. Children of parents with psychopathology may be at an increased risk of behavioral issues, psychiatric disorders, and lowered IQ due to an increased vulnerability associated with lower social class and gender [12]. Increased ACEs may impact the structural and emotional aspects of memory formation and retention [13]. Additionally, incarceration of a family member likely contributes to a wide range of developmental challenges for a child. Gifford et al. [14] found that children with a parent who is incarcerated are at an increased risk for maladjustment and mental illness (n=1,420). Among the 475 participants with any parental figure incarcerated, 39.9% reported experiencing any psychiatric diagnosis (anxiety, depressive disorder, ADHD, ODD, conduct disorder, and/or substance disorder) compared to 23% in the participants with no parental incarceration [14]. A responsive and cohesive family unit has been shown to contributes to positive effects in language and cognitive development, and also serves as a protective barrier to help children regulate emotions and behaviors during stressful situations [15]. With an incarcerated family member, a decreased ability to manage stressful situations may result in physiological developmental changes that negatively affect the body’s ability to regulate stressors due to dysregulation of cortisol production and stress response [13].

Parental divorce may impact a child’s life and lead to traumatic stress. Healthy coping mechanisms are essential for families to maintain or resume effective functioning following divorce. Deficits in family functioning and resiliency following a parental divorce may negatively impact children later in life by making them less likely to get help when needed. Gold et al. [5] found that parental remarriage, which may serve as a proxy for parental divorce, in a study population of 1,661 individuals aged 65 years and older is consistently associated with worse cognitive outcomes late in life (β=−0.11; 95% CI −0.20 to −0.03). The decline in socioeconomic status (SES) experienced after a parental divorce is considered one of the most important contributing factors in the decline of children’s cognitive functioning and perpetuates negative outcomes [16].

The CDC describes those with cognitive impairment as more likely to experience confusion, to have difficulty completing daily tasks, and to have a decreased ability to learn and make judgments, which may decrease their participation within society [17]. Numerous studies have also demonstrated that higher social engagement is correlated to higher cognitive functioning [18, 19]. Thus, not only are the individuals with SCD more likely to have social limitations, but also the increase in social limitations may exacerbate their SCD. While this is important among those with multiple ACEs, those reporting only one ACE of sexual abuse may be less likely to experience social limitations due to SCD. Frazier et al. [20] investigated the numerous factors that play a role in an increased likelihood of having positive life changes after sexual assault (n=171). Survivors who reported positive relations with social support and implementation of coping strategies were proven more likely to have positive changes and thus fewer limitations [20].

### Clinical implications and recommendations

This study’s findings correlated multiple ACEs with an individual’s reluctance to discuss SCD with his or her physician. This finding supports screening the use of interventions for individuals who may be at an increased risk of developing SCD. Whereas current screening guidelines from the US Preventive Services Task Force [21] found the evidence insufficient to assess the balance of benefits and harms of screening for cognitive impairment in older adults, our study found that individuals aged 65+ years old had the highest prevalence of SCD symptoms, targeting individuals aged 55–64 years old is important because we can prevent worsening symptoms, as suggested by the American Academy of Neurology [21, 22]. Thus, earlier SCD screening by healthcare providers in patients – especially within clinics regularly treating adults who have experienced early childhood trauma – may lead to earlier detection and improved healthcare outcomes among these individuals. Interventional and preventative programs targeting individuals reporting ACEs may lower the incidence and prevalence of SCD in future generations.

## Limitations

Limitations to this study include the subjectivity of self-reported ACEs and SCD questions within the BRFSS survey administration and possible misclassification of outcome variables (ACEs) given the length of time elapsed between childhood and responding to the survey questions. This time lapse may lead to inaccurate memories of ACEs experienced (recall bias) or omission of reporting of all ACEs – especially among individuals experiencing SCD. Another limitation is the measurement of SCD in the BRFSS SCD module because they do not assess the severity of symptoms, collect the age of onset of SCD, or indicate whether a clinical diagnosis was received [23]. Lastly, the associations found in this study are not adjusted for potential risk factors of SCD, such as brain trauma, stroke, alcohol use, etc., which may infer some vibration of effects [24]; however, because this study is correlational in nature, causality should not be inferred. The strengths of this study include the use of a large, nationally representative sample of the US population – in addition to including more state’s data on ACEs than any previous study. Future research endeavors may examine the potential impact of mediating variables such as adult depression between childhood trauma and SCD. Further, researchers may also examine how the implementation of preventative measures for childhood trauma impacts SCD in adulthood and how other potential risk factors of SCD may alter these outcomes.

## Conclusions

Our findings support previous research showing the association between SCD in adulthood and an accumulation of ACEs. Additionally, our novel approach found that individuals exposed to certain ACE categories – family incarceration, emotional abuse, sexual abuse, and parental divorce – were more likely to experience SCD compared to those with no ACEs. Our study demonstrates differential associations between individual ACE domains and their potential impact on an individual’s long-term cognitive functioning.

## Figures and Tables

**Table 1:** Categories of ACEs as defined by Felitti et al. [8].

Categories of adverse childhood experiences	
1.	**Mental illness in the household** (family mental illness) is defined as having a household member who was depressed, mentally ill, or attempted suicide
2.	**Substance abuse in the household** (family substance abuse) is defined as a member of the household being a problem drinker, alcoholic, or user of street drugs
3.	**Incarcerated household member** (family incarceration) is defined as having a household member who went to prison
4.	**Parental separation or divorce** is defined as having parents who were separated or divorced
5.	**Intimate partner violence** is defined as a member of the relationship being pushed, grabbed, slapped, had something thrown at them, kicked, bitten, hit with a fist, hit with something hard, repeatedly hit for over at least a few minutes, or ever threatened or hurt by a knife or gun by a member of the relationship
6.	**Emotional abuse** is defined as a parent or adult living in the home who swore, insulted, or spoke to the child in a way that made them afraid they could be physically hurt
7.	**Physical abuse** is defined as a parent or adult living in your home that pushed, grabbed, slapped, threw something at the child, or hit the child so hard that marks or injury occurred
8.	**Sexual abuse** is defined as an adult, relative, friend, or stranger at least five years older than the child who touched or fondled the child’s body in a sexual way or attempted to have any sexual intercourse with the child

**Table 2:** Demographics of BRFSS participants ages 45 years and older with and without memory loss^a^.

Characteristic	Experiencing cognitive decline (45+ years of age)	
	No	Yes
	n=160,345,	n=18,096,
	N=34,254,847	N=3,960,992
	n, %	n, %
**Age**		
45–54	32,336 (28.31)	3,134 (26.29)
55–64	44,827 (30.63)	4,812 (29.03)
65+	82,991 (41.06)	10,136 (44.67)
**Sex**		
Male	68,990 (46.72)	8,009 (44.89)
Female	91,164 (53.28)	10,073 (55.11)
**Race/ethnicity**		
White	129,389 (73.03)	14,352 (70.93)
Black	11,861 (11.28)	1,525 (12.32)
Asian	2,510 (2.30)	163 (1.60)
American Indian/Alaskan Native	2,533 (1.02)	411 (1.82)
Hispanic	9,471 (10.77)	1,053 (11.24)
Other	4,390 (1.60)	578 (2.08)
**Healthcare coverage**		
Yes	151,341 (92.41)	16,882 (91.17)
No	8,375 (7.59)	1,122 (8.83)
**Education**		
Did not graduate high	10,371 (12.36)	2,206 (22.24)
school		
High school graduate	41,445 (27.84)	5,464 (30.9)
Attended college but did not graduate	44,297 (30.59)	5,204 (28.67)
College or technical school graduate	63,501 (29.22)	5,160 (18.2)
**Adverse childhood experiences**		
Mean (SD)	1.44 (1.91)	2.61 (2.56)

a n=sample size; N = Population estimate, (#) are weighted percentages. The demographics including Race/Ethnicity, were self-reported by participants. BRFSS, Behavioral Risk Factor Surveillance System; SD, standard deviation.

**Table 3:** Associations of ACEs with reported frequent characteristics of cognitive decline.

	Logistic regression	
	OR (95% CI)	AOR (95% CI)
**Experienced confusion or memory loss**		
0 ACEs	1 [Ref]	1 [Ref]
1–2 ACEs	1.56 (1.41–1.72)	**1.59 (1.43–1.76)**
3+ ACEs	3.44 (3.11–3.81)	**3.58 (3.23–3.96)**
**Given up day-to-day household activities**		
0 ACEs	1 [Ref]	1 [Ref]
1–2 ACEs	1.04 (0.76–1.44)	0.99 (0.72–1.37)
3+ ACEs	1.92 (1.44–2.58)	**1.57 (1.16–2.12)**
**Needs help with day-to-day household activities**		
0 ACEs	1 [Ref]	1 [Ref]
1–2 ACEs	0.93 (0.66–1.31)	0.87 (0.61–1.23)
3+ ACEs	1.62 (1.16–2.27)	1.33 (0.96–1.86)
**Can get help when needed**		
0 ACEs	1 [Ref]	1 [Ref]
1–2 ACEs	0.75 (0.51–1.09)	0.82 (0.56–1.21)
3+ ACEs	0.53 (0.35–0.81)	**0.64 (0.43–0.93)**
**Limited social activities due to SCD**		
0 ACEs	1 [Ref]	1 [Ref]
1–2 ACEs	1.21 (0.88–1.67)	1.07 (0.77–1.49)
3+ ACEs	2.31 (1.68–3.17)	**1.71 (1.24–2.36)**
**Spoken to healthcare provider about SCD**		
0 ACEs	1 [Ref]	1 [Ref]
1–2 ACEs	0.77 (0.63–0.95)	**0.79 (0.65–0.97)**
3+ ACEs	0.59 (0.48–0.72)	**0.64 (0.52–0.79)**

BRFSS variables were binarily coded as frequent (usually or always) and infrequent (never, rarely, or sometimes). Adjusted models controlled for age, sex, race, healthcare coverage, and education. ACEs, adverse childhood experiences; AOR, adjusted odds ratio; BRFSS, Behavioral Risk Factor Surveillance System; CI, confidence interval; OR, odds ratio; SCD, subjective cognitive decline. Bolded values indicate significant findings.

**Table 4:** Associations of individuals reporting one ACE total within each ACE category with prevalence and associations with reported cognitive decline questions.

	Answering infrequently	Answering frequently	Total	Logistic regression	
	%	%	%	OR (95% CI)	AOR (95% CI)
**Experienced confusion or memory loss**					
No ACE	59.64	4.07	63.71	1 [Ref]	1 [Ref]
Family mental health	1.99	0.29	2.28	**2.09 (1.58–2.78)**	**2.32 (1.74–3.09)**
Family substance abuse	5.44	0.57	6.01	**1.54 (1.24–1.93)**	**1.55 (1.24–1.94)**
Family incarceration	0.37	0.06	0.43	2.22 (1.22–4.04)	**2.09 (1.11–3.94)**
Parental divorce	8.77	0.66	9.43	1.10 (0.87–1.39)	1.09 (0.86–1.38)
Intimate partner violence	1.39	0.11	1.5	1.19 (0.68–2.06)	1.08 (0.61–1.92)
Emotional abuse	7.65	0.66	8.32	1.27 (1.05–1.54)	**1.44 (1.19–1.74)**
Physical abuse	5.91	0.64	6.55	**1.58 (1.29–1.94)**	**1.51 (1.22–1.86)**
Sexual abuse	1.62	0.14	1.77	1.29 (0.94–1.77)	1.36 (0.99–1.88)
**Given up day-to-day household activities**					
No ACE	51.11	5.53	56.65	1 [Ref]	1 [Ref]
Family mental health	3.55	0.4	3.95	1.03 (0.52–2.04)	1.02 (0.50–2.07)
Family substance abuse	6.97	0.57	7.53	0.75 (0.40–1.41)	0.68 (0.36–1.3)
Family incarceration	0.64	0.13	0.77	1.93 (0.50–7.49)	2.16 (0.52–8.92)
Parental divorce	8.25	0.82	9.07	0.92 (0.57–1.50)	0.82 (0.49–1.37)
Intimate partner violence	1.43	0.12	1.55	0.77 (0.22–2.74)	0.82 (0.24–2.79)
Emotional abuse	9.29	0.72	10.01	0.72 (0.32–1.59)	0.77 (0.35–1.69)
Physical abuse	7.77	0.82	8.59	0.97 (0.56–1.67)	0.99 (0.57–1.71)
Sexual abuse	1.70	0.19	1.88	1.01 (0.30–3.38)	1.15 (0.35–3.78)
**Needs help with day-to-day household activities**					
No ACE	51.37	5.28	56.65	1 [Ref]	1 [Ref]
Family mental health	3.51	0.29	3.79	0.80 (0.37–1.71)	0.80 (0.36–1.78)
Family substance abuse	7.08	0.50	7.58	0.69 (0.32–1.48)	0.63 (0.30–1.32)
Family incarceration	0.65	0.12	0.77	1.86 (0.44–7.80)	1.99 (0.40–9.99)
Parental divorce	8.42	0.75	9.17	0.87 (0.51–1.47)	0.69 (0.39–1.24)
Intimate partner violence	1.53	0.04	1.57	0.28 (0.05–1.67)	0.26 (0.05–1.50)
Emotional abuse	9.14	0.66	9.8	0.70 (0.35–1.37)	0.79 (0.40–1.55)
Physical abuse	7.86	0.90	8.76	1.12 (0.65–1.92)	1.04 (0.56–1.91)
Sexual abuse	1.70	0.20	1.91	1.17 (0.38–3.61)	1.41 (0.47–4.27)
**Can get help when needed**					
No ACE	16.32	37.83	54.15	1 [Ref]	1 [Ref]
Family mental health	0.9	3.00	3.91	1.43 (0.56–3.68)	1.38 (0.59–3.23)
Family substance abuse	2.83	4.24	7.07	0.65 (0.34–1.23)	0.70 (0.36–1.37)
Family incarceration	0.31	1.11	1.42	1.54 (0.32–7.36)	1.84 (0.25–13.7)
Parental divorce	7.45	6.00	13.45	**0.35 (0.18–0.66)**	**0.53 (0.3–0.94)**
Intimate partner violence	0.39	0.57	0.96	0.63 (0.15–2.68)	0.83 (0.17–4.16)
Emotional abuse	3.95	5.29	9.24	0.58 (0.33–1.03)	0.54 (0.29–1.03)
Physical abuse	2.93	5.25	8.18	0.77 (0.46–1.29)	0.69 (0.39–1.19)
Sexual abuse	0.43	1.20	1.63	1.20 (0.38–3.83)	0.84 (0.22–3.13)
**Limited social activities due to SCD**					
No ACE	51.02	5.44	56.47	1 [Ref]	1 [Ref]
Family mental health	3.4	0.56	3.96	1.54 (0.75–3.18)	1.42 (0.64–3.16)
Family substance abuse	6.91	0.63	7.54	0.85 (0.44–1.65)	0.75 (0.36–1.54)
Family incarceration	0.5	0.23	0.72	4.29 (1.05–17.58)	3.40 (0.93–12.36)
Parental divorce	8.3	0.84	9.14	0.95 (0.59–1.54)	0.64 (0.37–1.11)
Intimate partner violence	1.46	0.11	1.57	0.70 (0.21–2.35)	0.80 (0.25–2.61)
Emotional abuse	8.42	1.51	9.94	1.68 (0.98–2.89)	1.68 (0.98–2.91)
Physical abuse	7.69	1.08	8.77	1.32 (0.71–2.43)	1.26 (0.70–2.27)
Sexual abuse	1.86	0.04	1.9	**0.20 (0.07–0.57)**	**0.24 (0.08–0.75)**
**Spoken to healthcare provider about SCD**					
No ACE	21.66	34.97	56.63	1 [Ref]	1 [Ref]
Family mental health	1.73	2.2	3.92	0.79 (0.47–1.32)	0.72 (0.44–1.17)
Family substance abuse	3.19	4.5	7.69	0.87 (0.58–1.32)	0.87 (0.58–1.30)
Family incarceration	0.39	0.38	0.77	0.60 (0.21–1.78)	0.61 (0.23–1.63)
Parental divorce	4.08	5.07	9.15	0.77 (0.49–1.2)	0.87 (0.57–1.33)
Intimate partner violence	0.79	0.76	1.55	0.59 (0.22–1.61)	0.80 (0.34–1.88)
Emotional abuse	3.99	5.93	9.92	0.92 (0.66–1.29)	0.97 (0.68–1.38)
Physical abuse	3.13	5.36	8.49	1.06 (0.74–1.53)	0.99 (0.66–1.48)
Sexual abuse	0.65	1.24	1.89	1.19 (0.69–2.05)	1.19 (0.69–2.07)

BRFSS variables were binarily coded as frequent (usually or always) and infrequent (never, rarely, or sometimes). Adjusted models controlled for age, sex, race, healthcare coverage, and education. ACEs, adverse childhood experiences; AOR, adjusted odds ratio; BRFSS, Behavioral Risk Factor Surveillance System; CI, confidence interval; OR, odds ratio; SCD, subjective cognitive decline. Bolded values indicate significant findings
